# Thoracoscopic treatment of mediastinal ectopic parathyroid adenomas: a Latinamerica experience case series and literature review

**DOI:** 10.1186/s13019-024-02694-y

**Published:** 2024-04-04

**Authors:** Chavez Karla Veronica, Chavez-Tostado Mariana, Peña-Rivera Adriana Graciela, Cervantes-Perez Gabino, Bolaños-Morales Francina Valezka

**Affiliations:** 1 Surgery Department, Medica Sur Hospital, Mexico city, Mexico; 2 Department of Human Reproduction, University of Guadalajara, Health Sciences University Center, Guadalajara, Mexico; 3 Internal Medicine Department. “Fray Antonio Alcalde” Hospital, Guadalajara, Mexico; 4 Thoracic surgery department, Instituto Nacional de Enfermedades Respiratorias, Mexico City, Mexico

**Keywords:** Hyperparathyroidism, mediastinal ectopic parathyroid adenoma, Video-assisted thoracoscopic surgery

## Abstract

**Background:**

Hyperparathyroidism (HPT) is a disease caused by hypersecretion of one or more parathyroid glands, it can be associated with ectopic mediastinal parathyroid glands (MEPA) in 2% of cases. The use of video-assisted thoracoscopic surgery (VATS) for the surgical resection of these glands is a safe, cost-effective, and low morbidity option for patients with MEPA. We report a case series of patients with this disease managed with VATS, the first in Mexico and Latinamerica.

**Methods:**

From 2008 to 2022, a retrospective study involving patients with MEPA and treated by VATS approach was performed in a tertiary hospital in Mexico city. Relevant biochemical and clinical variables such as imaging studies, pre and postoperative laboratory results, surgical strategy, outcomes and pathological analysis were analyzed.

**Results:**

Four cases of mediastinal parathyroid adenomas causing HPT were included. All patients were female with a median age of 52.5 years-old (range 46–59 years), half of the patients had primary HPT and the others tertiary HPT after kidney transplant. 75% of cases had a MEPA in the medium mediastinum, all had a preoperative positive SPECT-CT 99mTc Sestamibi scan. Mean preoperative PTH was 621.3pg/mL (182-1382pg/mL). All patients successfully underwent parathyroidectomy with a VATS approach, no deaths were reported.

**Conclusions:**

VATS is a minimally invasive surgery that provides adequate access to mediastinal located glands, optimal visualization of mediastinal structures and has a high resection success rate with less complications and morbidity than open approaches.

## Background

Hyperparathyroidism (HPT) is a clinical disorder characterized by an inappropriately elevated paratohormone (PTH) due to hypersecretion of one or more parathyroid glands, that may develop secondary hypercalcemia and other metabolic disturbances. This disease may present with various signs and symptoms such as nephrolithiasis, osteopenia and osteoporosis, depression, mental numbness, loss of appetite, nausea, vomiting, constipation among others. The abnormal secretion of PTH is most commonly caused by a single parathyroid gland adenoma in 85% of the cases, in the other 15% is due to multiple gland hyperplasia (15–20%) [1] or rarely from a parathyroid carcinoma (< 1%). HPT occurs in both genders equally around the sixth decade [2]. World HPT prevalence is estimated at 1 in every 500 women and 1 in every 2000 men [3].

Mediastinal ectopic parathyroid adenomas (MEPA) are rare tumors, constituting only 1–2% of all parathyroid adenomas. These glands are inferior to the sternal notch and their location may vary from the superior mediastinum to the pericardium and diaphragm [4]. The first report of a mediastinal parathyroid adenoma was in 1932 by Churchill in the patient Captain Charles E. Martell, who had 6 prior cervical explorations for until an ectopic gland was found in the superior mediastinum [5]. Before the introduction of VATS, MEPA were usually resected by thoracotomy or a median sternotomy, currently with the daily use of minimally invasive surgery, VATS is being adopted as the procedure of choice.

In this manuscript we report a case series of patients with MEPA treated with VATS, along with a literature review.

## Materials and methods

A retrospective study was performed in patients admitted with a diagnosis of MEPA in a reference Hospital in Mexico City from 2008 to 2022. The inclusion criteria were adults with hyperparathyroidism diagnosis, who presented MEPA on imaging studies. Exclusion criteria were loss of follow-up or incomplete clinical records. Their clinical and demographic variables including comorbidities, clinical history, laboratory results, surgical technique and operative details, post operative evolution, length of hospital stay, and clinical outcome were recorded and analyzed. Non-parametric univariate (descriptive) statistics were used when necessary, such as median and range. General features were summarized in tables.

### Surgical technique

Patients under general anesthesia are placed in a lateral decubitus contralateral to the location of the gland, selective intubation is carried with a double-lumen endotracheal tube; for glands in the anterior mediastinum a right lung intubation is performed. If the ectopic parathyroid is on the left side (aortopulmonary window), selective intubation is performed and the left lung is excluded as the right side continues to be ventilated. If the ectopic parathyroid is on the right side, with the left double lumen tube, the left lung continues to be ventilated and the right one is collapsed; everything is visualized under direct vision with the bronchoscope.

Depending on the localization of the MEPA the thoracic ports are placed in order to obtain the best visualization and working space; if it is located in the anterior mediastinum, it is approached in supine decubitus, the first 10.5mm port is placed in the 5th intercostal space in the middle midclavicular line, another 5 mm submammary port in the midclavicular line and another one in the anterior axillary line at the level of the 7th-8th intercostal space, in this port the camera is placed to have a panoramic view. If we want to introduce some thoracoscopy forceps a field expander is placed in the 5th space were we place the first port.

If it is in the middle or posterior mediastinum, the patient is placed in lateral decubitus, on the left side when it is located behind the trachea or esophagus, and on the right side when it is in the aortopulmonary window. A 10.5 mm port is placed in the 5th intercostal space middle axillary line, the camera is introduced towards the diaphragm to place an anterior port at the level of the 7th space anterior in the axillary line and another 10.5 mm port is placed, the last port is placed in the 7th posterior space.

If it is in the aortopulmonary window, the patient is placed in the right decubitus. The first 10.5 mm port is placed in the 5th intercostal space in the middle axillary line, where the camera is introduced to place an anterior port at the level of the 7th space anterior axillary line and to place the 3rd port in the 7th posterior space under direct view.

No carbon dioxide is insufflated. Once we have access to the thoracic cavity, the mediastinal pleura is opened and the adenoma is then identified, dissected, and extracted with special care of not tearing the gland´s capsule or fragment it. We only use bipolar or ultrasonic energy to avoid thermal damage to the adjacent structures. After assuring hemostasia, the pneumothorax is aspirated and a closed pleural drainage such as a Blake® (Ethicon, Inc. Somerville, New Jersey) is left until it has an output of less than 0.01 ml/kg/day or 500 ml/day.

Intra-operative PTH is measured according to the Miami protocol [6], postoperative calcium levels and X-Ray are measured daily. Patients are fed once they are awake and usually are discharged the following days.

## Results

**Fig. 1:**
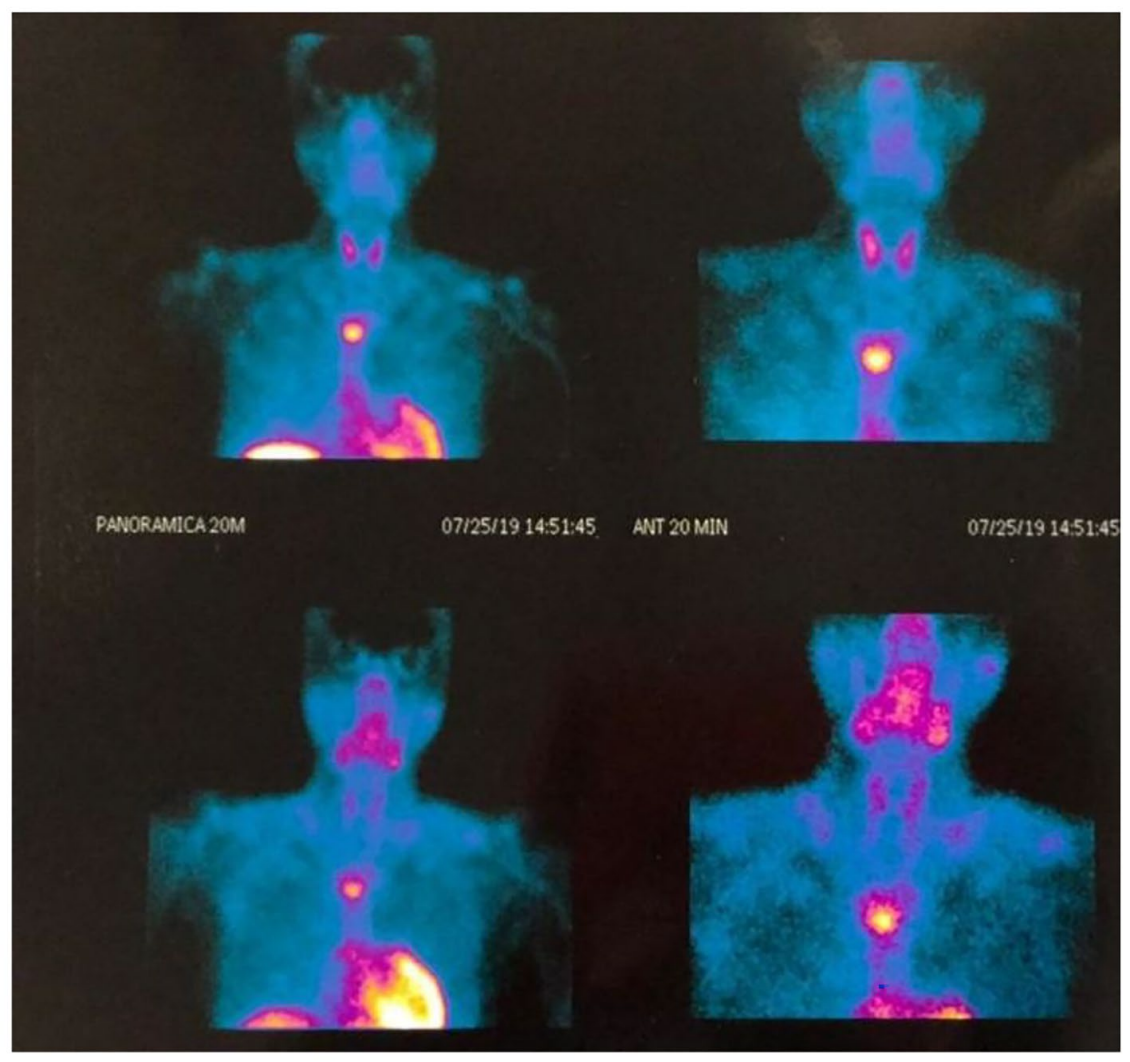
Results of a 99mTc Sestamibi SPECT scan in a patient with a MEPA in the medium mediastinum

**Fig. 2:**
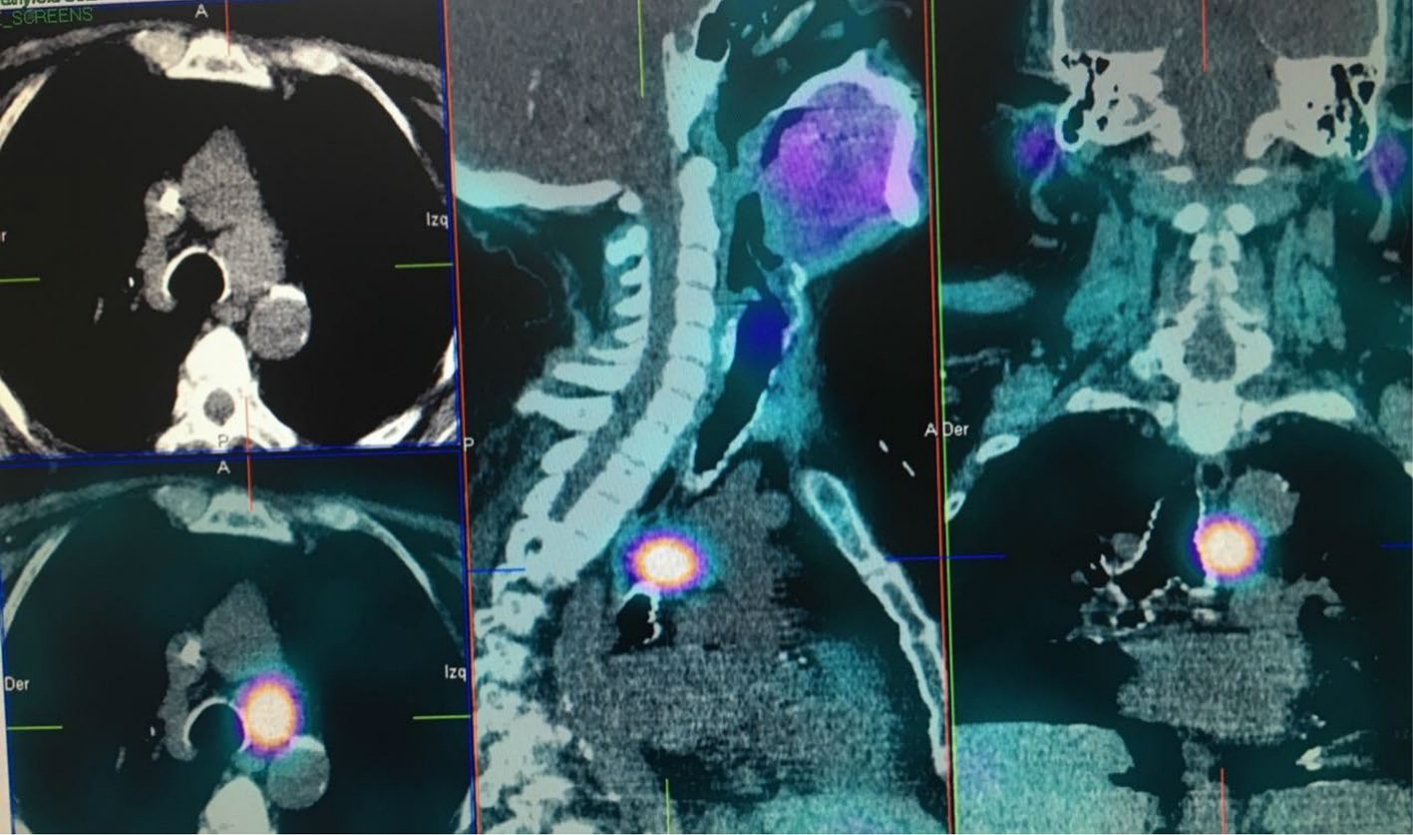
SPECT-CT scan with 99mTc Sestamibi in a patient with a MEPA in the aorto-pulmonary window

**Fig. 3:**
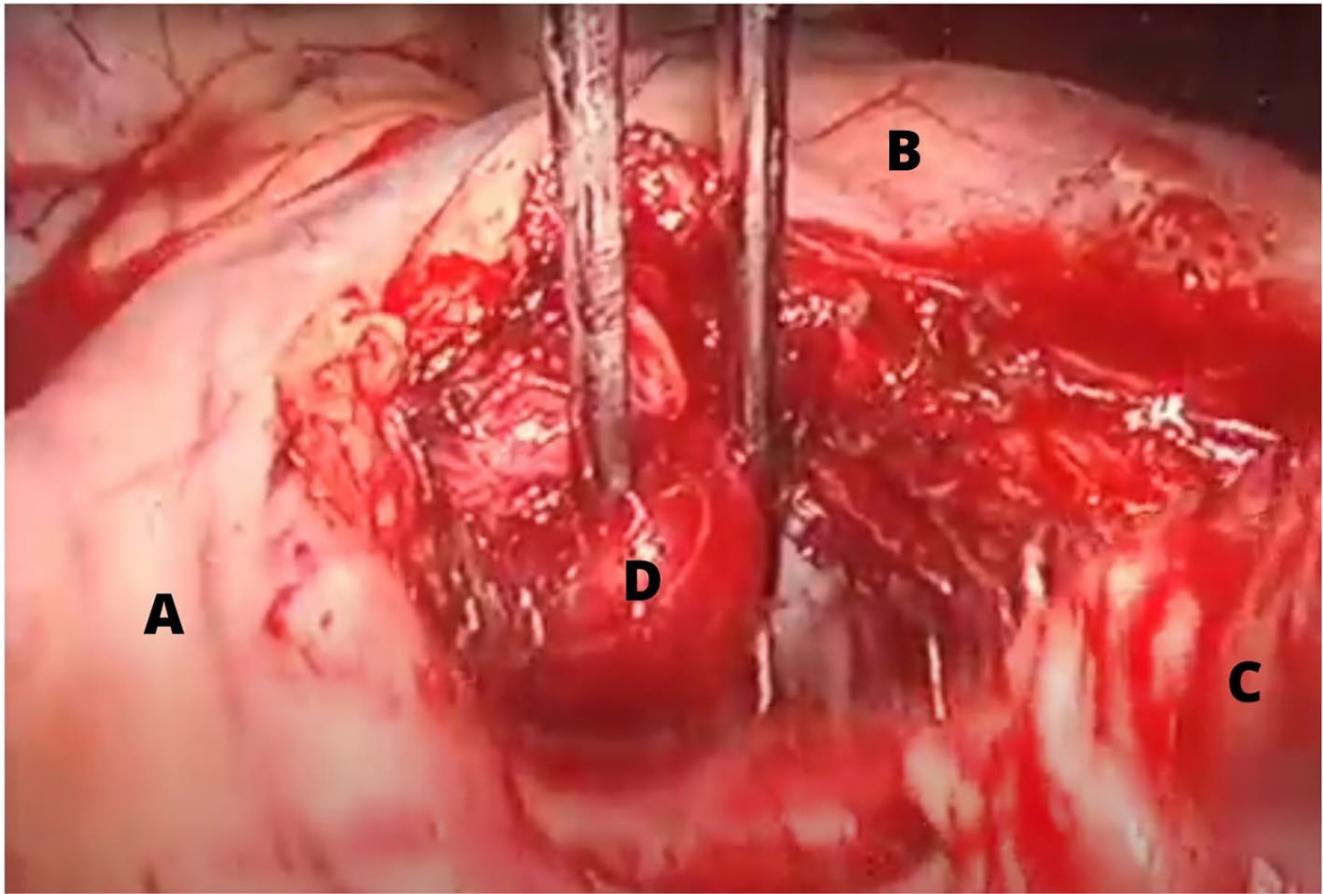
VATS resection of a MEPA located in the aorto-pulmonary window. **A**: aortic arch, **B**: thoracic aorta, **C**: pulmonary vein, **D**: parathyroid adenoma

Four cases of mediastinal parathyroid adenomas causing HPT were included. All these patients were female with a median age of 52.5 years (range 46–59 years), half of the patients had a primary HPT and the other had tertiary HPT after kidney transplant due to end stage renal disease, 75% had a history of nephrolithiasis. Laboratory test showed a mean preoperative PTH of 621.3pg/mL (182-1382pg/mL) and a mean calcium of 10 mg/dL (7.02–15.1 mg/dL) other clinical and biochemical features of these patients are displayed in Table 1. All patients had a positive 99mTc Sestamibi scan (Fig. 1), in two cases the MEPA were located in the medium mediastinum, one of them in the aorto-pulmonary window (Figs. 2 and 3) and only one in the anterior mediastinum.

**Table 1:** Clinical and biochemical features of patients with MEPA

Variable	Case 1	Case 2	Case 3	Case 4
Age (years)	59	51	54	46
Gender	Female	Female	Female	Female
Pre-operative corrected calcium (mg/dl)	9,7	15,1	7,02	10
Pre-operative PTH (pg/dl)	182	302,9	1382	300
Tumor size after removal (mm)	18 × 14	Multifragmented	29 × 35 × 27	30 × 15 × 10
Post-operative calcium (mg/dl)	9	10,40	8,30	8
Post-operative PTH (pg/dl)	372,2	74,50	25,6	100
Parathyroid auto transplantation	NO	Left arm	Left arm	NO
Surgical time (min)	180	160	140	120
Hospital Stay (days)	16	25	16	7

All patients underwent a VATS approach, the mean operative time was 148 min (range 120–180 min). One patient had an incidental vascular injury to an inferior bronchial artery branch, which was repaired with no serious implications. After surgery, one patient developed metabolic disturbances due to hungry bone syndrome and had a longer hospital stay of 25 days. The mean hospital stay was 16 days, no mortality was recorded. The pathology analysis of all patients reported parathyroid adenomas, concordantly to the preoperative diagnosis.

## Discussion

Surgical resection is the definitive treatment of patients with HPT. Bilateral neck exploration is currently the gold standard for the surgical treatment of patients with a cervical parathyroid adenoma [7]. In these cases, a focused single gland parathyroid exploration can be performed with other adjuvants as the use of intraoperative PTH [8] however, approximately 16% of patients with HPT have an ectopic parathyroid gland and up to 2% of hyperfunctioning parathyroid adenomas are not accessible by a standard cervical surgical approach [9].

Ectopic localization of the parathyroid glands is attributed to an abnormal migration during embryogenesis or as the result of primary mediastinum development [10]. Because the inferior parathyroid glands undergo more extensive migration during embryogenesis, they are more likely to be found in abnormal ectopic locations [11]. These include the thyroid-thymic ligament, the retro/paraoesophageal space, the mediastinum, intrathymic or intrathyroidal, within the carotid sheath and/or a high-undescended cervical position [12].

The possibility of an ectopic localization is why preoperative localization studies for HPT must be performed in all patients, including neck ultrasound, Computed Tomography (CT) scan, Magnetic Resonance Imaging (MRI) or Single Photon Emission Computed Tomography (SPECT-CT) as Scintigraphy with 99mTc Sestamibi which displays 100% sensitivity and 97.4% positive predictive value for the detection of ectopic parathyroid adenoma [13] in HPT patients. Recent reports also describe the usefulness of 18 F-flurocholine in PET for patients with occult adenomas [14]. In our series, all patients had a positive SPECT-CT with a single MEPA, which dictated the surgical approach. Recently, indocynanine green has increased in popularity and use in the field of endocrine surgery, however, its usefulness in identifying mediastinal ectopic parathyroid adenomas has not yet been demonstrated; currently there is only one case report of its use in a carotid sheath adenoma [15]; the same is true for autofluorescence and methylene blue infusion [16], therefore their routine use is not yet recommended.

The clinical presentation of a MEPA is commonly more dramatic, they often have a longer standing disease, previous cervical explorations, and a delayed diagnosis. They tend to be more hypercalcemic, with a more pronounced bone reabsorption and kidney stones [4]. Rarely, they present with thoracic bleeding due to a ruptured gland hematoma or with symptoms due to compression of adjacent structures such as stridor or dysphagia [17].

Depending on how deep in the mediastinum the gland is located a transcervical, trans-sternal or thoracic approach is necessary: for glands in the superior mediastinum (above the aortic arch) the transcervical approach is the procedure of election, as the upper mediastinum is easily reached through a retrosternal dissection. For the medium mediastinum and lower located MEPA, a medium sternotomy or thoracotomy is needed. Nowadays, the video-assisted thoracoscopic approach for the surgical resection of MEPA is the preferred one, because of its numerous benefits over traditional open procedures, which can be associated with significant complications including phrenic and recurrent laryngeal nerve injuries, innominate vein laceration, wound infections, mediastinitis and death [4].

The first report of the use of a thoracoscopic approach to resect a MEPA was described by Prinz et al. in 1994 [18]. VATS is a feasible and safe approach for resecting these glands, with an overall success ratio of 98–100% [4]. It has several advantages over traditional open approaches as any other minimally invasive techniques, such as less bleeding, less operative time, less pain, better cosmesis, less intrahospital stay, more rapid recovery [10, 19], and allows better visualization of the tumor due to the magnification of structures [16] with the endoscopic lens. According to Masatoshi [20], all glands under the aortic arch can be resected with VATS, but it must be performed by a trained thoracic surgeon with VATS training, a vast anatomy knowledge together with an experienced group of endocrinologist, endocrine surgeons and anesthesiology to avoid potential catastrophic complications(16). In our case series, all patients were eligible to this approach because of their adenoma localization in the medium or lower anterior mediastinum. All the procedures were successfully performed by a trained thoracic surgeon in thoracoscopic surgery, with only one complication due to bleeding of an accessory inferior bronchial artery that was repaired during surgery without conversion.

Several reports of the use of VATS are described in the literature, however, more randomized, high-quality studies are needed to determine if VATS can be the gold standard approach for MEPA.

## Conclusions

MEPA are rare tumors that present with hyperparathyroidism in patients with non-cervical localized adenomas, they are difficult to diagnose and to treat. Open surgery can be used to achieve a successful resection but has a high morbidity. VATS is a minimally invasive surgery that provides adequate access to mediastinal located glands, optimal visualization of mediastinal structures and has a high resection success rate with less morbidity than open approaches, hence it should be considered the first line approach for the resection of MEPA.

## Data Availability

Not applicable.

## Abbreviations

HPT: Hiperparathyroidism
PTH: Paratohormone
MEPA: Mediastinal Ectopic Parathyroid Adenona
VATS: Video-Assisted Thoracoscopic Surgery
SPECT-CT: Single Photon Emission Computed Tomography
